# Time-lapse image analysis for whole colony growth curves and daily distribution of the cell number per colony during the expansion of mesenchymal stem cells

**DOI:** 10.1038/s41598-019-53383-z

**Published:** 2019-11-14

**Authors:** Mitsuru Mizuno, Hisako Katano, Yuri Shimozaki, Sho Sanami, Nobutake Ozeki, Hideyuki Koga, Ichiro Sekiya

**Affiliations:** 1 Center for Stem Cell and Regenerative Medicine, Tokyo Medical and Dental University, 1-5-45, Bunkyo-ku, Yushima, Tokyo Japan; 2 Research & Development Center, Dai Nippon Printing Co., Ltd., Tokyo, Japan; 3 Department of Joint Surgery and Sports Medicine, Graduate School of Medical and Dental Sciences, Tokyo Medical and Dental University, Tokyo, Japan

**Keywords:** Phase-contrast microscopy, Software, Mesenchymal stem cells, Mesenchymal stem cells

## Abstract

Mesenchymal stem cells from the synovium (synovial MSCs) are attractive for cartilage and meniscus regeneration therapy. We developed a software program that can distinguish individual colonies and automatically count the cell number per colony using time-lapse images. In this study, we investigated the usefulness of the software and analyzed colony formation in cultured synovial MSCs. Time-lapse image data were obtained for 14-day-expanded human synovial MSCs. The cell number per colony (for 145 colonies) was automatically counted from phase-contrast and nuclear-stained images. Colony growth curves from day 1 to day 14 (for 140 colonies) were classified using cluster analysis. Correlation analysis of the distribution of the cell number per colony at 14 days versus that number at 1–14 days revealed a correlation at 7 and 14 days. We obtained accurate cell number counts from phase-contrast images. Individual colony growth curves were classified into three main groups and subgroups. Our image analysis software has the potential to improve the evaluation of cell proliferation and to facilitate successful clinical applications using MSCs.

## Introduction

Mesenchymal stem cells (MSCs) are an attractive cell source for cell therapies. Among the many MSC types, primary synovial MSCs are useful for cartilage regeneration in clinical situations^1^ because synovial MSCs can be easily cultured from synovium and have a high chondrogenic potential^2^. Synovial MSCs are currently undergoing clinical trials to promote healing after meniscus repair^3^. For clinical use, sufficient numbers of MSCs must be prepared within a limited culture period and passages; however, this is not always achieved due to donor variations. Detailed analysis of the cell culture process may help to resolve this problem.

Time-lapse analysis is one method for analyzing cell cultures, so a fully automatic cell counting system based on phase-contrast images would be advantageous. However, phase- contrast images have a critical problem of noise arising from the presence of red blood cells or tissue debris^4^. We have addressed this limitation by the development of novel image analysis software that can distinguish living cells from other debris. Our first purpose was to compare the numbers of synovial MSCs counted by our novel image analysis software in phase-contrast images and in nuclear-stained images.

During the primary culture of MSCs, nucleated cells divide, form cell colonies, and expand. However, the MSCs do not show uniform colony-forming abilities^5–7^ due to differences in their differentiation ability^8^. Interestingly, even when MSCs in the same population are cultured, the initial cell density affects the colony size, resulting in differences in cartilage differentiation ability^9^. Colony information could therefore predict the characteristics of the MSCs. Furthermore, since cell growth is the sum of individual colony growth curves, the analysis of individual colony growth curves can advance the understanding of cell growth. Our second purpose was therefore to analyze individual colony growth curves of synovial MSCs using our novel image analysis software. In a clinical setting, obtaining a set number of MSCs during a fixed period requires some ability to predict cell yields in the early stages of culture, because yields can be improved by options such as adding serum, changing media, and replating the cells. Our novel image analysis software enabled us to determine the distribution of cell numbers per colony based on time-lapse images taken during the culture period. Our third purpose was to investigate whether analysis of the cell number per colony distribution could determine which day of culture would best predict the cell yields at 14 days.

## Methods

### Synovial MSCs

This study was approved by the Medical Research Ethics Committee of Tokyo Medical and Dental University, and all study subjects provided written informed consent. Human synovium was harvested from the knees of nine donors (mean age: 76.2 ± 5.3 years) with osteoarthritis during total knee arthroplasty. The synovial tissue was digested in a solution of 3 mg/mL collagenase (Sigma-Aldrich Japan, Tokyo, Japan) at 37 °C^10^. After 3 hours, the digested cells were filtered through a 70-μm cell strainer (Greiner Bio-One GmbH, Frickenhausen, Germany) and cultured in α-Minimal Essential Medium (Thermo Fisher Scientific, Waltham, MA, USA) supplemented with 1% antibiotic-antimycotic (Thermo Fisher Scientific) and 10% fetal bovine serum (Thermo Fisher Scientific) in a humidified cell culture chamber (Tokai Hit, Fujinomiya, Japan) set at 37 °C with 5% CO_2_, 20% O_2_ and 75% N_2_. The cells were counted with an automated cell counter (Luna-FL; Logos Biosystems, Annandale, VA, USA) in a disposable cell counting plate to determine the number of nucleated cells.

### Time-lapse imaging

After enzyme digestion, nucleated synovial cells were seeded at 20 cells/cm^2^ in 6-well plates and cultured for 14 days. Whole wells were scanned by time-lapse microscopy using a computerized multi-area time-lapse imaging system (IX83ZDC; Olympus, Tokyo, Japan). Images were acquired every 6 hours for 14 days and were reconstructed as a time-lapse movie using image analysis software (Dai Nippon Printing Co., Tokyo, Japan).

### Cell recognition from phase-contrast images

The software that we have introduced here allowed the recognition of cells from phase-contrast images. The images that exhibited halation at the cell edges and whose centers were darker than the surrounding background were first optimized for image analysis. Next, the gradation of the background brightness was adjusted. The cell shape was then restored from the phase- contrast images using an image filter^11^, and the cell shape was identified by binarizing the restored shape (Fig. S1). Objects that were in the same position in several frames were defined as living cells, while objects that moved rapidly, such as debris, were defined as image noise (Fig. S2).

### Colony definition

A colony was defined as a cell population containing a minimum of 4 cells within a diameter of 320 μm (80 pixel). The identified colonies were subsequently characterized as either “merged” or “non-merged.” If the closest cell spacing between colonies was less than 100 μm, both colonies were considered merged colonies and were excluded from the analysis. Conversely, a non-merged colony was defined as a non-merged colony.

### Equivalence evaluation between phase-contrast and fluorescence cell images

Synovial MSCs at 4 days and 14 days were stained with DAPI (Wako, Osaka, Japan) diluted 1:5000 in phosphate-buffered saline (PBS; Thermo Fisher Scientific). Phase-contrast images and nuclear-stained images were then obtained from two wells derived from two donors, and the cell number per colony was counted for 145 colonies with our image analysis software. The coefficient of determination (R^2^) between the cell number from the phase-contrast images and the cell number from the nuclear-stained images at day 4 and day 14 was calculated by regression analysis before the data were converted to a logarithmic scale.

### Classification of colony growth curves

Consecutive growth measurements for cell number per colony were analyzed from day 1 to day 14 for 140 colonies. The differences were large, so the cell numbers per colony were converted to their square root values to reduce the difference. Cluster analysis was performed using R software and the furthest neighbor method^12^.

### Morphological properties of the colony

The long axis of cells was calculated using the length of the long axis of the ellipse that has the same normalized second central moments as the region of interest (Fig. S3). The mean of all the long axes of cells forming the colony was defined as a data of the colony.

The distance between cells was established by first determining the center of gravity by averaging the X and Y values of individual pixel coordinates in each recognized cell area. The cell centers of gravity were then connected using the Delaunay triangulation method, which create triangles connecting all the points to maximize the smallest angle (Fig. S3)^13^. The average of all distances between cells within a colony was then calculated.

### Correlation analysis

Each colony (n = 140) was monitored and the cell number per colony was counted at 1–14 days. The coefficient of determination (R^2^) between the cell number per colony at 14 days and that at 1–14 days was calculated by regression analysis.

### Statistical analysis

Mean differences were evaluated by analysis of variance using Prism 7 software (GraphPad Inc., La Jolla, CA, USA). Two-tailed P values < 0.05 were considered significant.

### Ethics approval and consent to participate

This study was approved by the institutional review board of Tokyo Medical and Dental University (Reference Number: M2017-142), and was carried out in accordance with the Helsinki Declaration. Written informed consent was obtained from all participating patients.

## Results

### Image analysis software for counting cell number per colony

**Figure 1:**
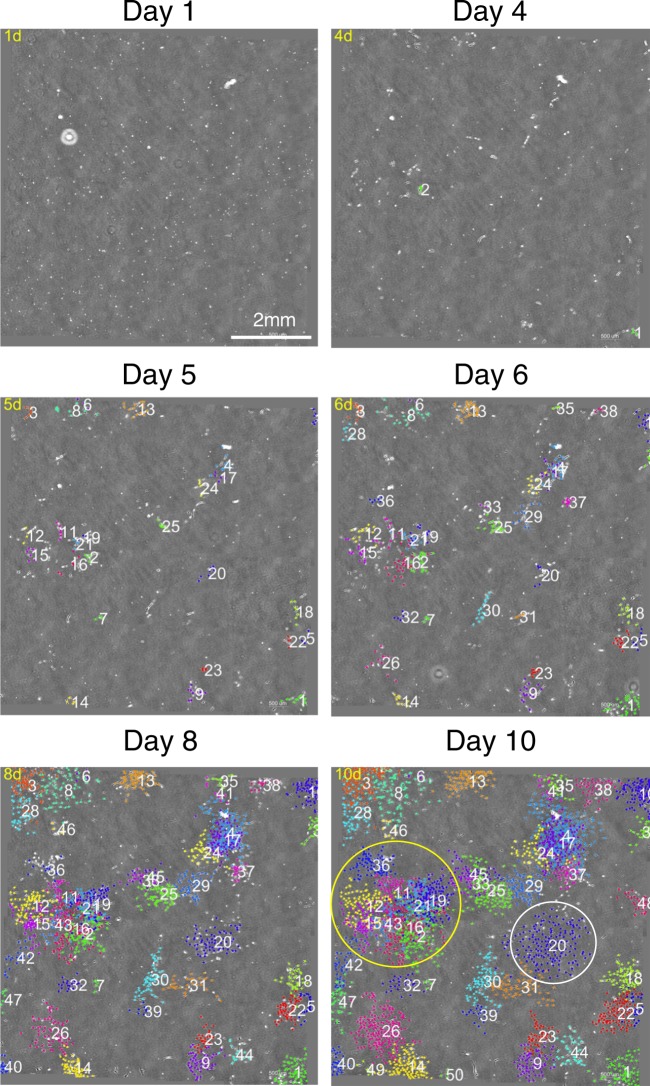
Multi-colored images of synovial MSC colony formation. Synovial MSCs were plated at 20 cells/cm^2^ and cultured. When single cells had proliferated to yield four cells, the colonies were colored and counted using our software. White circles show single cell-derived colonies, and yellow circles show a single colony consisting of nine single cell-derived colonies.

Synovial MSCs plated at 20 cells/cm^2^ formed colonies and proliferated (Supplementary Movie 1 and Supplementary Fig. S4). We developed a software program that colored and numbered each colony when a single cell increased to yield more than four cells (Supplementary Movie 2 and Fig. 1). On day 10, we observed a colony derived from a single cell and one consisting of multiple single cell-derived colonies (Fig. 1). Our software was able to distinguish proliferated cells from debris (Fig. S2).

**Figure 2:**
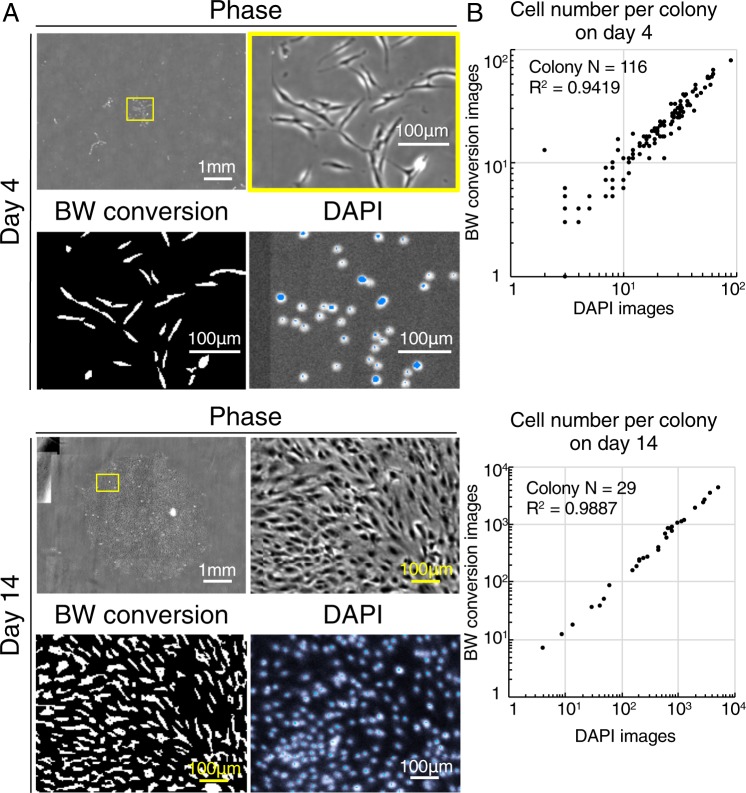
The cell number per colony evaluated from images converted to black and white (BW) and DAPI images. (**A**) Black and white images and DAPI signal images of synovial MSCs. Cells were plated at 20 cells/cm^2^ and cultured. Images were converted to BW using our software, and the DAPI signal appears as blue. (**B**) Correlation between the cell number per colony from BW converted images and DAPI signal images analyzed by Spearman’s correlation coefficient.

Our software converted phase-contrast images to black and white images (Fig. 2A), followed by counts of the cell number per colony. The accuracy of this method was verified by comparison with cell counts obtained using synovial MSCs stained with 4′,6-diamidino-2-phenylindole (DAPI; Fig. 2A) on days 4 and 14. The two sets of counts were highly correlated (Fig. 2B).

### Growth curves of cell number per colony and their classification

**Figure 3:**
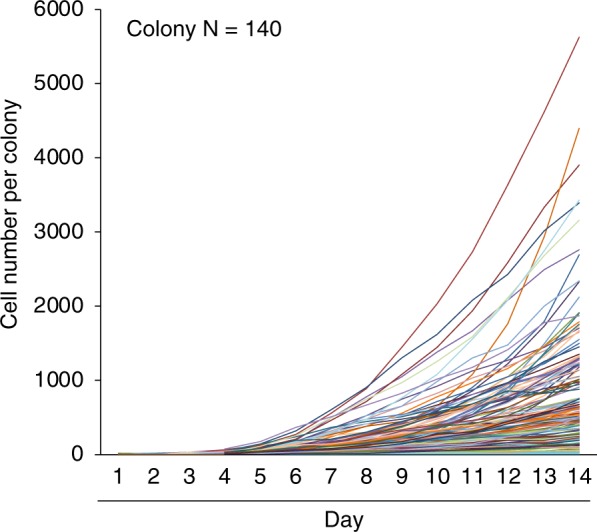
Individual growth curves of the cell number per colony in MSC colonies. Synovial MSCs were plated at 20 cells/cm^2^ and cultured for 14 days. In total, 140 colonies were analyzed using our software.

**Figure 4:**
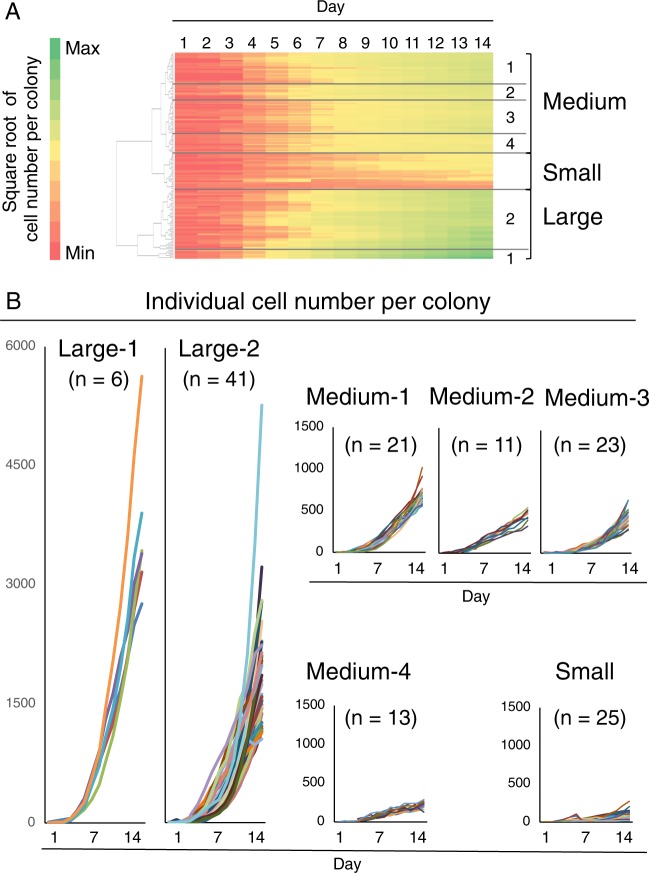
Classification of MSC colony growth curves by the cell number per colony. (**A**) Cluster analysis based on the growth curve of the cell number per colony, which was classified into large 1,2, medium 1–4, and small groups. (**B**) Individual growth curve of the cell number per colony in each group.

Our software generated individual growth curves of cell number per colony for 140 colonies (Fig. 3). Cluster analysis showed that the growth curves could be classified as large-1, large-2, medium-1 to medium-4, and small groups (Fig. 4A). The cell number per colony on day 14 was the highest in the large-1 group and the lowest in the small group (Fig. 4B). Among the medium groups, the rank order of cell number per colony on day 14 were medium-1 > medium-2 ≈ medium-3 > medium-4. The medium-2 and -3 groups were distinguished from each other by their log-growth phases, which occurred earlier in the medium-2 group than in the medium-3 group.

**Figure 5:**
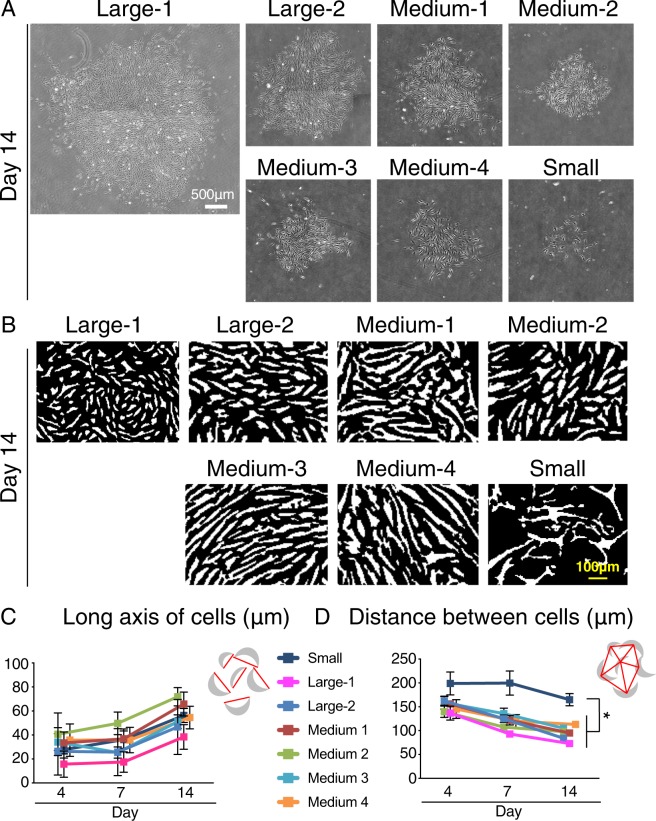
Analysis of cell colonies in the large 1,2, medium 1–4, and small groups. Synovial MSCs were plated at 20 cells/cm^2^ and cultured for 14 days. (**A**) Representative colony images. (**B**) Representative black and white converted images in each group. (**C**) Quantitative analysis of the cell long axis in each colony. (**D**) Quantitative analysis of the distance between cells in each colony. *P < 0.05 by Kruskal Wallis test and Dunn’s multiple comparisons test.

The large 1 group had the largest colonies on day 14, whereas the small group had the smallest (Fig. 5A). Black and white converted images clarified the morphological differences between the cell colonies (Fig. 5B). No differences were noted in the long axis of cells among the different colony groups, but the distance between the cells was significantly wider in the small colony group than in the other groups (Fig. 5C,D).

### Predicting the distribution of cell number per colony

**Figure 6:**
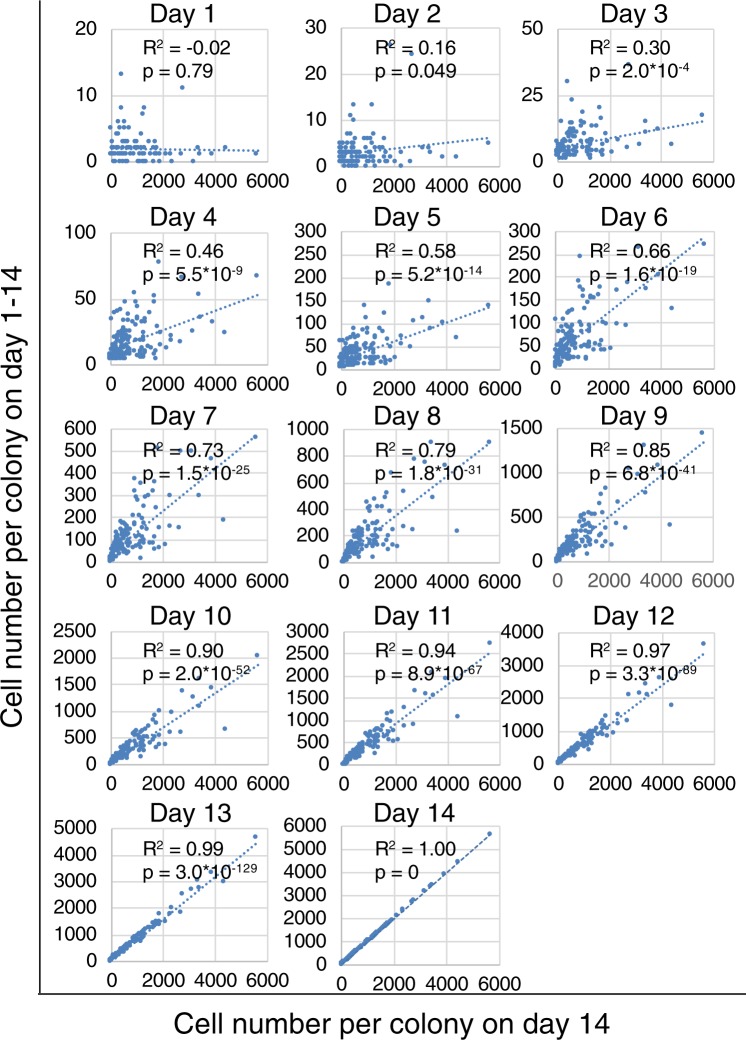
Correlation analysis of the cell number per colony on day 14 and days 1–14. Synovial MSCs were plated at 20 cells/cm^2^ and cultured for 14 days. The cell number per colony was counted daily using our software.

We predicted the distribution of the cell number per colony by performing a correlation analysis between the cell number per colony on day 14 and on days 1–14. The coefficient of determination (R^2^) by regression analysis increased with culture period (Fig. 6). The R^2^ value exceeded 0.7 on day 7 and beyond.

## Discussion

We developed software to analyze phase difference images from time-lapse data. Conventional cell image analysis is limited by image noise, such as tissue fragments and blood cells, which can lead to misrecognition of the cells^14^. In previous research, image noise was filtered based on the size of the targets^15,16^. However, primary cultures of synovial MSCs contain noise as large as the cells themselves, so this noise cannot be removed based on size alone. We solved this problem by analyzing the dynamics of individual targets in continuous observation.

We were able to obtain accurate cell number counts from phase-contrast images. Several reports have described methods for counting the number of cells from cultured cell images. Nuclear staining^17^ and genetic editing of the cells^18^ are two methods that have generated accurate cell number measurements, however, these are invasive methods and are not suitable for clinical applications. Other methods have also been reported for cell detection using phase contrast-images. For example, Buggenthin *et al*. compared image analysis and manual counting in cultures of hematopoietic stem cells (HSCs) and obtained an accuracy of over 80%^15^. This previous report is important, as the number of cells was automatically counted from the phase- contrast image; however, HSCs maintain a stable circular shape and are easier to count than cells with fibroblast-like morphologies. Similarly, Suga *et al*. were able to estimate the number of cells per colony from the size of the colony by comparison with manual counts, and they showed an R^2^ value greater than 0.9^19^. An accurate measurement will only be possible if the shape of all colonies is uniform. In the current study, the accuracy seemed to be low when the number of cells per colony was less than 20 cells. However, our study is valuable in that our software directly counted the number of fibroblast-like cells from the phase- contrast images with high accuracy when the number of cells per colony was more than 20 cells.

The final number of cells per colony may depend on the initial location of the cells in the seeding process. For instance, if several cells are initially close to each other, the growing colonies will be restricted by the expansion of the adjacent colonies; therefore, the final size and number of cells will effectively depend on the cell’s initial location. Conversely, a cell initially located far from any other cell will not encounter these constraints, and its colony will grow extensively. Another reason underlying the larger cell distance in the “small colony group” may be that this group of colonies ceases growing earlier due to their initial cell locations. Alternatively, the final number of cells per colony may depend on the cell itself. We have shown that the proliferative activity of synovial MSCs differed depending on the original location in synovium^4^. In other cells, the cell morphology is correlated with cellular properties, such as proliferation and differentiation^20–22^, as well as metabolic activities^23,24^. Further detailed morphological analysis should reveal differences in the colony growth of synovial MSCs.

In clinical situations that require the use of autologous cells, a method for non-invasive yield prediction would be useful in the early culture period^25,26^. We thought that the distribution of the cell number per colony data would be a good predictor of final yield since it can be obtained using phase-contrast image data. In this study, the coefficient of determination between the cell number per colony on day 14 and days 1–14 increased with culture period and exceeded 0.7 on day 7, indicating that the distribution of the cell number per colony on day 7 could be an indicator of the final yield at 14 days, since a strong correlation is generally considered to exist when R^2^ exceeds 0.7^27^. Verification of the practical usefulness of this indicator will await further prospective studies.

In the present study, we analyzed only synovial MSCs and did not examine MSCs derived from other sources, such as bone marrow and adipose tissue. All MSCs have their own specific properties, but their cell morphology during the colony formation seems to be similar to that observed with synovial MSCs. Further examination should therefore indicate whether our image analysis software will also be applicable to other MSC types.

The synovium, bone marrow, and fat cells all have colony-forming abilities and trilineage (osteogenic, adipogenic, and chondrogenic) differentiation potential^2,7,28^. Although their properties differ, all these cells exhibit fibroblastic-like morphology^2^. Individual analysis of each of these cell types can lead to new insights regarding their potential usefulness in cell-based therapies.

The present study had some limitations. First, this software is under development and is specialized for use with only specific fibroblast-like cells. Therefore, the use of phase-contrast images for identification of other cell types should be customized according to their specific morphology and images using alternative microscopes. We are currently developing new recognition technologies for various other cell types. Second, we plated synovial nucleated cells at 20 cells/cm^2^, which is a considerably lower initial cell density than is used in clinical situations (approximately 5000 cells/cm^2^). We chose this density because we wanted to avoid contact between the colonies to facilitate our analysis. This condition differed from that used at the clinical level; however, to the best of our knowledge, this is the first study to provide a systematic documentation of colony growth of MSCs during expansion. Our investigation will help to reveal cell proliferation mechanisms and aid in the successful clinical application of MSCs.

## Supplementary information

Supplementary Information

Supplementary Movie 1

Supplementary Movie 2

## Data Availability

All data supporting the results can be found in this manuscript and as Supplemental Data. Data requests can be addressed to the corresponding author.
