# Supplementary material for: Time-lapse image analysis for whole colony growth curves and daily distribution of the cell number per colony during the expansion of mesenchymal stem cells
Source: Sci Rep. 2019 Nov 14;9:16835. doi: 10.1038/s41598-019-53383-z (PMC6856116; doi:10.1038/s41598-019-53383-z)

Time-lapse image analysis for whole colony growth curves and daily distribution of the cell number per colony during the expansion of mesenchymal stem cells

Mitsuru Mizuno[1], Hisako Katano[1], Yuri Shimozaki[2], Sho Sanami[2],

Nobutake Ozeki[1], Hideyuki Koga[3], Ichiro Sekiya[1]*

[1]Center for Stem Cell and Regenerative Medicine, Tokyo Medical and Dental University, 1-5-45, Bunkyo-ku, Yushima, Tokyo, Japan

[2]Research & Development Center, Dai Nippon Printing Co., Ltd., Tokyo, Japan

[3]Department of Joint Surgery and Sports Medicine, Tokyo Medical and Dental University, Tokyo, Japan

*Correspondence information

Ichiro Sekiya, MD, PhD.

Director and Professor, Center for Stem Cell and Regenerative Medicine,

Tokyo Medical and Dental University, 1-5-45 Yushima, Bunkyo-ku, Tokyo 113-8510, Japan.

Phone: +81-3-5803-4017, fax: +81-3-5803-0192, e-mail: sekiya.arm@tmd.ac.jp

Supplementary Information

Supplementary Movie 1. Time-lapse imaging during colony formation of synovial MSCs. After collagenase digestion, the synovial nucleated cells were plated at 20 cells/cm^2 in a 6-well plate. For time-lapse imaging, the images were captured every 6 h for 14 days, for a total of approximately 500,000 phase-contrast images.

Supplementary Movie 2. Multi-colored images obtained during synovial MSC colony formation. When single cells proliferated to yield four cells, the colony was colored and numbered using our software.

Supplementary Figures

Figure S1. Cell recognition from phase-contrast images. (A) Original phase-contrast image. (B) Image after brightness adjustment. (C) Restored image obtained using a filter. (D) BW conversion image.

Figure S2. The debris removal process in phase-contrast image analysis. Debris was identified as objects that moved rapidly (indicated by the red arrow) or that did not change form (representative examples are indicated by yellow arrows) during continuous monitoring.

Figure S3. In this representative colony, the individual cells are indicated by red pixels. Double-ended arrows indicate the long axis of the cells and the yellow number is the length of the long axis (μm). The blue lines indicate the distance between cells and blue number is the distance between the cells (μm). In this example, the average length of the long axis was 37.9 μm and the average distance between cells was 88.4 μm.

Figure S4. Colony formation of primary synovial MSCs. Single MSCs proliferated to form colonies composed of approximately 1000 cells.

Figure S1, Cell recognition from phase-contrast images

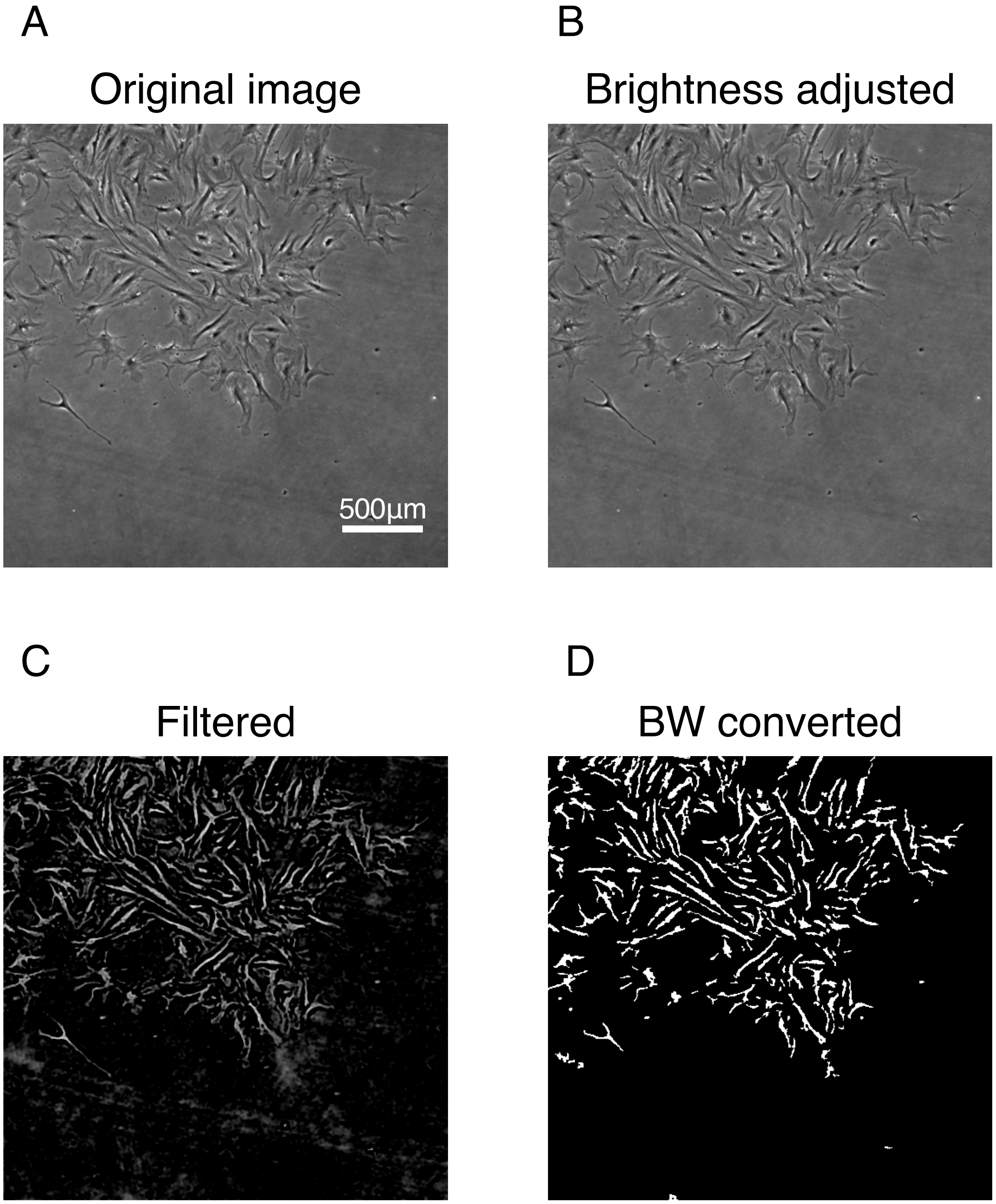

Figure S2, Removal of image noise

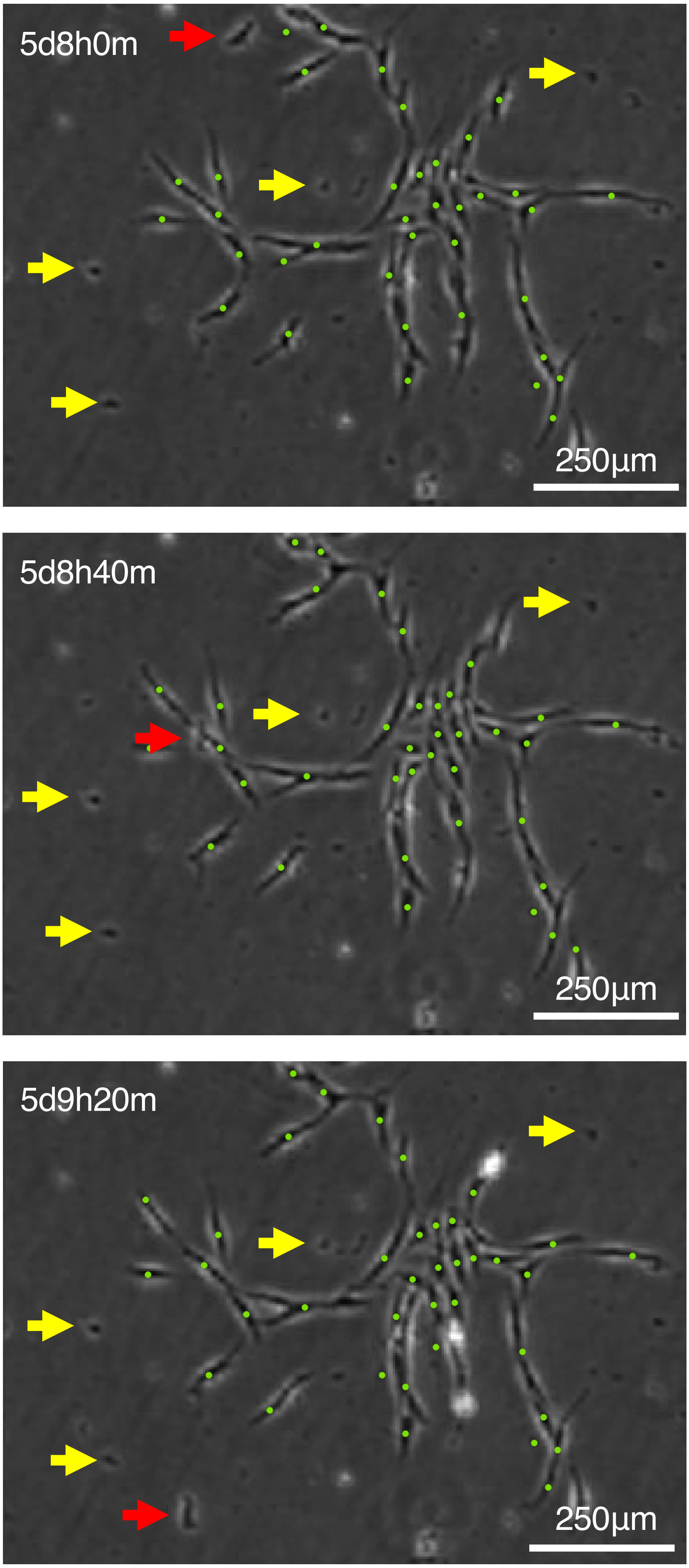

Figure S3, Morphological properties

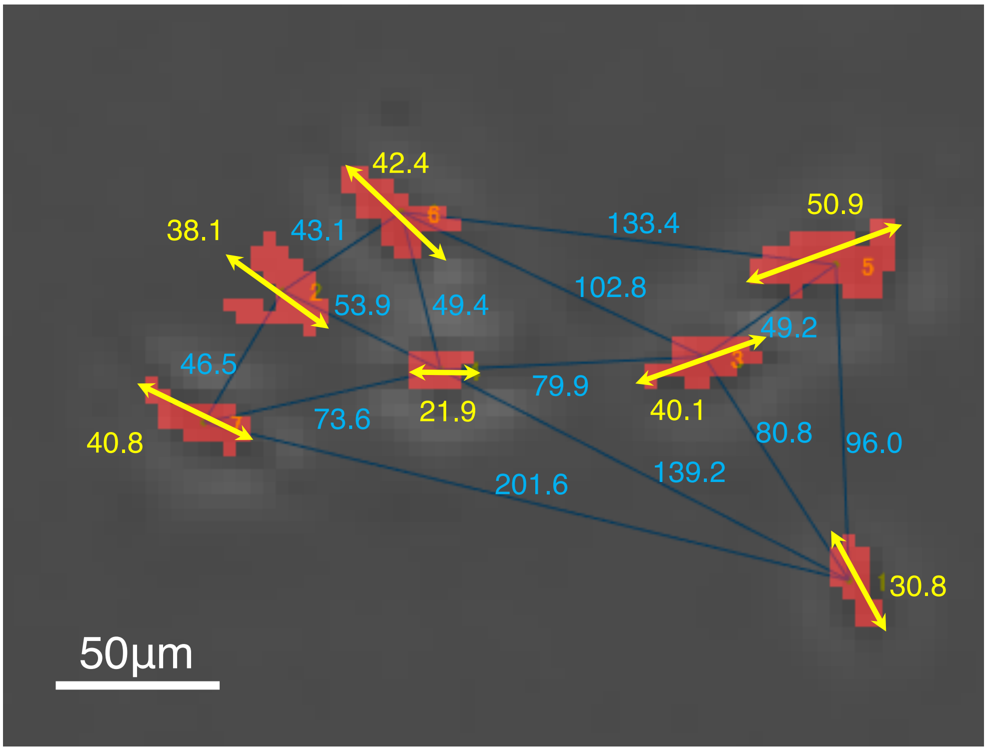

Distance between cells in colony: 88.4µm
Long axis of cells in colony: 37.9µm

Figure S4, Colony growth of primary synovial mesenchymal stem cells

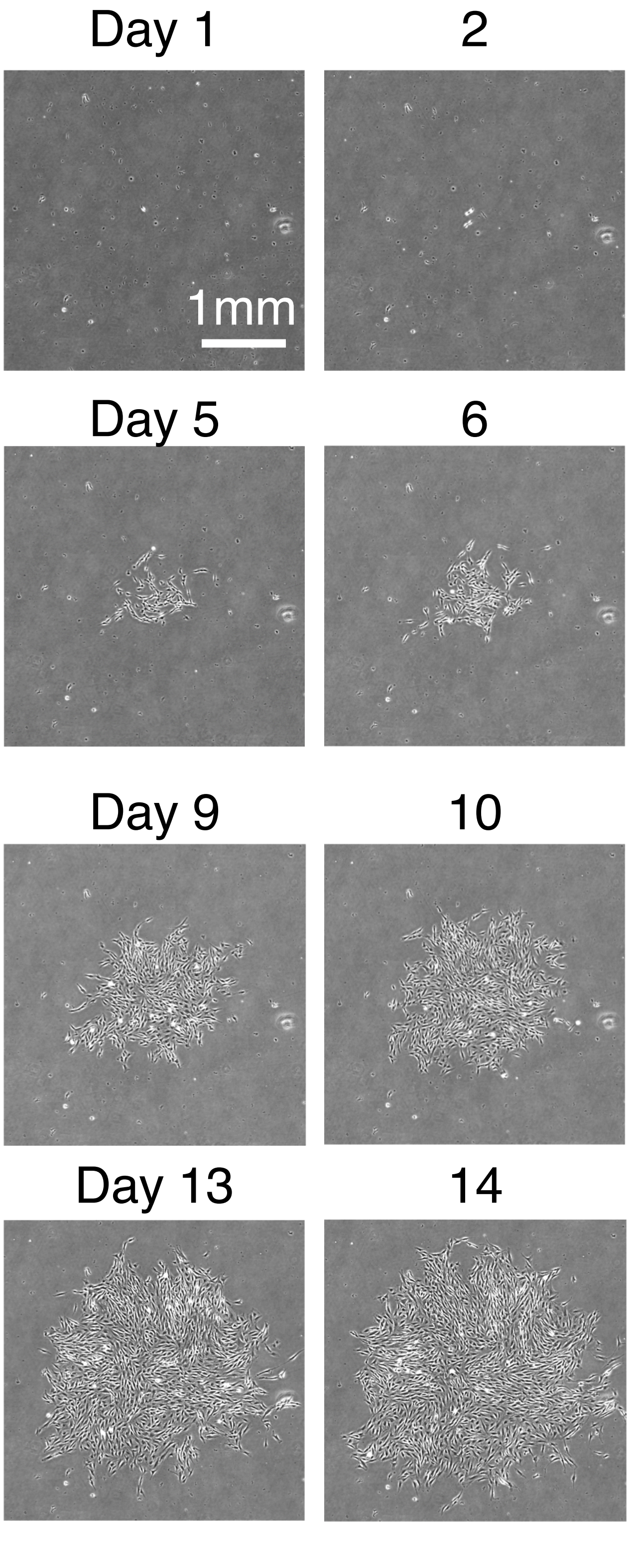

Supplement: Supplementary file 1 — Supplementary Information [file 41598_2019_53383_MOESM1_ESM.pdf]
